# Molecular insights into the maturation of phosphodiesterase 6 by the specialized chaperone complex of HSP90 with AIPL1

**DOI:** 10.1016/j.jbc.2022.101620

**Published:** 2022-01-21

**Authors:** Ravi P. Yadav, Kimberly Boyd, Nikolai O. Artemyev

**Affiliations:** 1 Department of Molecular Physiology and Biophysics, The University of Iowa Carver College of Medicine, Iowa City, Iowa, USA; 2 Department of Ophthalmology and Visual Sciences, The University of Iowa Carver College of Medicine, Iowa City, Iowa, USA

**Keywords:** photoreceptor, phosphodiesterase 6, phototransduction, chaperone, HSP90, protein folding, AhR, aryl hydrocarbon receptor, AIP, AhR-interacting protein, AIPL1, aryl hydrocarbon receptor-interacting protein-like 1, AIPL1-FKBP, FKBP-domain of AIPL1, AIPL1-TPR, TPR-domain of AIPL1, BLI, Biolayer Interferometry, BME, β-mercaptoethanol, CTD, C-terminal domain, DMAG, 17-dimethylaminoethylamino-17-demethoxygeldanamycin, DSS, disuccinimidyl suberate, FKBP, FK506-binding protein, PDE6, Phosphodiesterase 6, SA, streptavidin, SEC, size-exclusion chromatography, TCEP, tris(2-carboxyethyl)phosphine, TPR, tetratricopeptide repeat

## Abstract

Phosphodiesterase 6 (PDE6) is a key effector enzyme in vertebrate phototransduction, and its maturation and function are known to critically depend on a specialized chaperone, aryl hydrocarbon receptor-interacting protein-like 1 (AIPL1). Defects in PDE6 and AIPL1 underlie several severe retinal diseases, including retinitis pigmentosa and Leber congenital amaurosis. Here, we characterize the complex of AIPL1 with HSP90 and demonstrate its essential role in promoting the functional conformation of nascent PDE6. Our analysis suggests that AIPL1 preferentially binds to HSP90 in the closed state with a stoichiometry of 1:2, with the tetratricopeptide repeat domain and the tetratricopeptide repeat helix 7 extension of AIPL1 being the main contributors to the AIPL1/HSP90 interface. We demonstrate that mutations of these determinants markedly diminished both the affinity of AIPL1 for HSP90 and the ability of AIPL1 to cochaperone the maturation of PDE6 in a heterologous expression system. In addition, the FK506-binding protein (FKBP) domain of AIPL1 encloses a unique prenyl-binding site that anchors AIPL1 to posttranslational lipid modifications of PDE6. A mouse model with rod PDE6 lacking farnesylation of its PDE6A subunit revealed normal expression, trafficking, and signaling of the enzyme. Furthermore, AIPL1 was unexpectedly capable of inducing the maturation of unprenylated cone PDE6C, whereas mutant AIPL1 deficient in prenyl binding competently cochaperoned prenylated PDE6C. Thus, we conclude neither sequestration of the prenyl modifications is required for PDE6 maturation to proceed, nor is the FKBP-lipid interaction involved in the conformational switch of the enzyme into the functional state.

Cyclic nucleotide phosphodiesterases of the sixth family (PDE6) are the effector enzymes in both rod and cone photoreceptor cells, whereby light-induced activation of PDE6 generates a hyperpolarizing electrical response propagated to downstream retinal neurons (1, 2). Rod PDE6 is composed of a catalytic PDE6AB heterodimer and two copies of the small inhibitory Pγ subunits, which are displaced from the catalytic core during phototransduction (3). Cone PDE6 is a homodimer of catalytic PDE6C subunits each associated with the cone-specific Pγ subunit (3). The PDE6 genes each encode the C-terminal CAAX box for posttranslational prenylation: PDE6A and PDE6B subunits are farnesylated and geranylgeranylated, respectively, whereas PDE6C is geranylgeranylated (4). The lipid modifications of the PDE6 catalytic subunits serve at least two primary functions. Prenyl moieties enable proper trafficking of PDE6 from the photoreceptor inner segment, a site of protein synthesis, to the outer segment, a site of phototransduction (5). Secondly, they provide for the photoreceptor membrane attachment of PDE6 that is important for efficient signal amplification and deactivation (6). Phosphodiesterase 6 deficits often underlie retinal diseases: mutations in the PDE6A and PDE6B subunits, as well as in rod Pγ, can cause recessive retinitis pigmentosa (7, 8, 9) and autosomal dominant congenital stationary night blindness (10); and mutations in their cone counterparts, PDE6C and cone Pγ, cause autosomal recessive achromatopsia (11, 12, 13, 14). Both rod and cone PDE6 rely on a specialized chaperone, aryl hydrocarbon receptor (AhR)-interacting protein-like 1 (AIPL1), for the maturation that is needed for correct function (15, 16, 17, 18). Consistent with AIPL1 serving as a chaperone, its knockout in mice leads to drastic reduction of both the levels and activity of PDE6, as well as severe and rapid degeneration of the retina (15). Mutations in AIPL1 lead to a severe form of Leber congenital amaurosis, a major cause of blindness in children (19, 20, 21). Despite the key roles that PDE6 and AIPL1 play in human vision and photoreceptor health, the mechanism whereby AIPL1 facilitates the maturation of PDE6 to its functional form is virtually unknown.

AIPL1 shares a high sequence identity (∼50%) with AhR-interacting protein (AIP), which serves as cochaperone of HSP90 during the maturation of AhR (19, 22). AIP and AIPL1 both contain an N-terminal FK506-binding protein (FKBP) domain and a C-terminal tetratricopeptide repeat (TPR)-domain. Tandem FKBP and TPR domains are also present in FKBP51/52 proteins, which serve as HSP90 cochaperones in the maturation of steroid hormone receptors (23). The FKBP-domain of AIPL1 (AIPL1-FKBP) encloses a unique site for prenyl lipid binding, which is thought to be critical to the protein function as a PDE6 chaperone (24, 25). The prenyl moieties, thus, may represent critical tethers for PDE6 anchoring to AIPL1. The TPR-domain of AIPL1 (AIPL1-TPR), similarly to the well-known interactions of TPR-domain proteins with HSP90 (26), binds to the C-terminal signature sequence MEEVD of HSP90 (27, 28). Moreover, the FKBP-domains of AIP and FKBP51 were shown to interact with HSP90 raising the possibility that AIPL1-FKBP contributes to the protein interface with HSP90 (29, 30, 31). Thus, given the ability of AIPL1-FKBP to bind prenyl modifications of PDE6, a ternary chaperone-client complex of HSP90/AIPL1 with nascent PDE6 can be envisioned as an intermediate in PDE6 maturation. Consistent with the notion that PDE6 is a client for the HSP90/AIPL1 complex, prolonged pharmacological inhibition of HSP90 reduced the stability of PDE6 in mouse retina (32). However, direct evidence that a complex of HSP90 and AIPL1 is required for correct folding of PDE6 has been lacking. Recent studies reported increased levels of cGMP in cells cotransfected with PDE6 and AIPL1 (33, 34). This effect was reduced or absent when the cells were cotransfected with PDE6 and AIPL1 variants that are deficient in HSP90 binding (33, 34). The drawback of relying on measurements of cGMP levels is that they reflect the balance of cGMP synthesis by guanylyl cyclase and breakdown by PDE6 activity. Because functional PDE6 is expected to reduce cellular levels of cGMP, the origin of the observed changes in cGMP levels is unclear. Here, we characterized the interaction of HSP90β (hereafter called HSP90) with AIPL1 and provided direct evidence that the HSP90/AIPL1 complex is required for the maturation of PDE6. We show that AIPL1 preferentially binds to the closed HSP90 state with a stoichiometry of 1:2. HSP90 inhibitor 17-dimethylaminoethylamino-17-demethoxygeldanamycin (DMAG) potently blocks the expression of functional PDE6C in HEK293T cells, whereas AIPL1 mutations disrupting its interaction with HSP90 markedly diminish the chaperone activity of the complex. Furthermore, using a knock-in mouse model and the heterologous expression system, we investigated the role of PDE6 prenyl modifications in the HSP90/AIPL1-dependent maturation of PDE6. Surprisingly, our analysis suggests that the ability of the chaperone complex to induce an active conformation of PDE6 does not absolutely require the enzyme prenyl modifications or the prenyl-binding capacity of AIPL1.

## Results

### AIPL1 preferentially interacts with the closed conformation of HSP90

**Figure 1:**
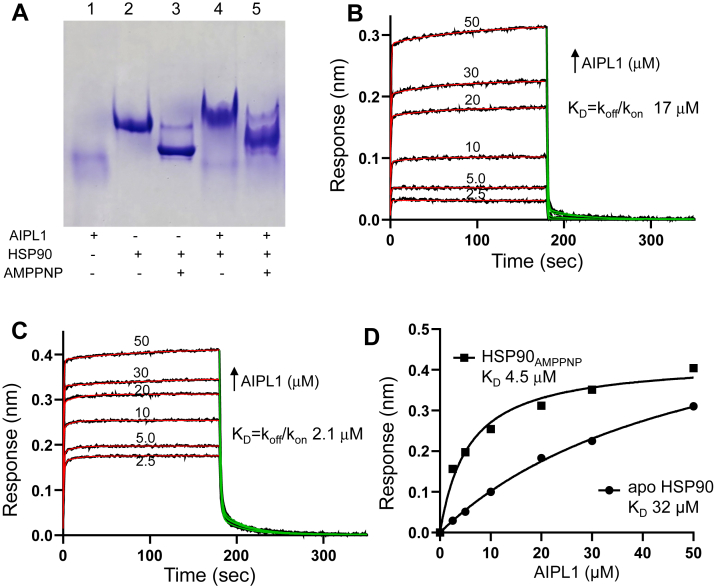
**Interaction of HSP90 with AIPL1.***A*, native gel analysis shows that apo HSP90 (lane 2) after incubation with 2 mM AMPPNP for 2-h at 37 °C in the presence of 500 mM NaCl adopts a faster migrating closed state (lane 3). In the presence of AIPL1, both apo HSP90 and HSP90_AMPPNP_ form slower migrating HSP90/AIPL1 complexes (lanes 4 and 5). *B* and *C*, kinetics of association and dissociation for AIPL1 and Avi-tagged apo HSP90 (*B*) or HSP90_AMPPNP_ (*C*) coupled to a streptavidin biosensor as determined using BLI. The processed data curves are *black* and the nonlinear regression fits from the 1:1 binding model are *red* (association) and *green* (dissociation); *B*, k_on_ = 7.1 ± 0.3 × 10^4^ M^−1^s^−1^; k_off_ = 1.2 ± 0.1 s^−1^; *C*, k_on_ = 1.5 ± 0.4 × 10^5^ M^−1^s^−1^; k_off_ = 0.32 ± 0.10 s^−1^ (Mean ± SE). *D*, the steady state binding curves obtained from data in (*B* and *C*); For n = 2 experiments, apo HSP90 K_D_ = 33 μM (range 32–34 μM); HSP90_AMPPNP_ K_D_ = 4.8 μM (range 4.5–5.2 μM). AIPL1, aryl hydrocarbon receptor-interacting protein-like 1; BLI, Biolayer Interferometry.

HSP90 chaperones exist mainly as homodimers in conformations ranging from a closed ATP-bound state to open/V-shaped and open-extended states for the apo protein (35, 36). We investigated interactions of the full-length mouse AIPL1 (aa 1–328) with the apo- and AMPPNP-bound states of HSP90. Transition of HSP90 from the open apo state to the closed state after incubation with AMPPNP was confirmed by the increased protein mobility during native gel electrophoresis (Fig. 1*A*). Addition of AIPL1 to the apo- or the AMPPNP-bound HSP90 led to a shift toward a slower migrating protein complex, suggesting that the cochaperone can bind to both HSP90 states. To assess these interactions quantitatively, we conducted Biolayer Interferometry (BLI) assays using Avi-tagged HSP90 attached to the streptavidin (SA) sensor. These experiments revealed fast association/dissociation kinetics of AIPL1 binding to apo HSP90, yielding a K_D_ (k_off_/k_on_) of ∼17 μM (Fig. 1*B*). A K_D_ of 32 μM was indicated by the steady-state analysis, reflecting a relatively weak binding of AIPL1 to the open HSP90 state (Fig. 1*D*). The binding assay between AIPL1 and the closed HSP90_AMPPNP_ state indicated a significantly higher-affinity interaction and a kinetic and steady-state K_D_ values of ∼2.1 μM and ∼4.5 μM, respectively (Fig. 1, *C* and *D*).

FKBP51 has been recently shown to preferentially and asymmetrically interact with the closed HSP90 conformation with a 1:2 stoichiometry (37). The asymmetry and stoichiometry of the FKBP51/HSP90 complex were underlined by the previously uncharacterized interaction of the TPR helix 7 extension with the C-terminal domain (CTD) of HSP90 (37). To examine a potential role of the AIPL1-TPR helix 7 extension in binding to HSP90, we truncated 12 C-terminal residues in mouse AIPL1 (AIPL1_1–316_) (Fig. S1*A*). Using the BLI assay, the affinity of AIPL1_1–316_ for the closed HSP90 state was significantly lower than that of the wt AIPL1 (K_D_ 27 μM) (Fig. S1*B*). The unique “insert” region of the FKBP domain and its α3-helix were shown to be critical for the ability of AIPL1 to chaperone PDE6 (Fig. S1*A*) (38). From the binding assay, the impact of the α3-helix deletion on the interaction of AIPL1 with HSP90 was relatively modest (Fig. S1*B*).

To probe the stoichiometry of the HSP90_AMPPNP_/AIPL1 complex, the reconstituted proteins were cross-linked with disuccinimidyl suberate (DSS) or glutaraldehyde. The main product of the HSP90 dimer self-crosslinking migrated on an SDS-PAGE gel at ∼210 kDa, apparently because of extensive modification of the protein (Figs. S2 and S3). The cross-linking of AIPL1 to HSP90 by both DSS and glutaraldehyde yielded a major product of ∼250 kDa (Figs. S2 and S3) and a minor product of ∼290 kDa, which are consistent with the complexes containing one and two molecules of AIPL1 per HSP90 dimer, respectively. Analysis of the crosslinked products by mass photometry revealed the masses of both crosslinked HSP90/AIPL1 complexes more accurately as 228 and 268 kDa (Fig. S3). These masses closely match the predicted molecular weight values. We surmise from this analysis that AIPL1 binds to HSP90 predominantly at a 1:2 stoichiometry, but a second AIPL1 molecule can bind with low affinity. Alternatively, at a high concentration AIPL1 may weakly dimerize (Fig. S3*A*), and crosslinking of such a dimer to HSP90 dimer would also yield a minor 268 kDa product.

### HSP90 inhibitor DMAG potently attenuates expression of functional PDE6 in cell culture

**Figure 2:**
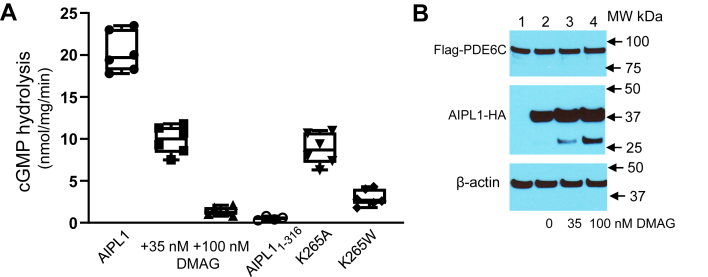
**Inhibition of HSP90 and AIPL1 mutations disrupting its binding to HSP90 impede maturation of PDE6 in transfected HEK293T cells.***A*, cGMP hydrolysis in lysates of HEK293T cells cotransfected with PDE6C, Pγ, AIPL1 (in the absence and presence of HSP90 inhibitor DMAG), or AIPL1 mutants (untransfected control is subtracted). Because Pγ is the inhibitory subunit of PDE6, the samples were treated with trypsin to selectively remove Pγ before assay. *Whiskers* represent minimum and maximum. *Boxes* represent interquartile range. *Line* represents the median and *dots* represent data points. The PDE6 activity values are (nmol•min^−1^• mg^−1^): AIPL1, 20.3 ± 1.0; 35 nM DMAG, 9.8 ± 0.7 (n = 6); 100 nM DMAG, 1.4 ± 0.2 (n = 6); AIPL1_1–316_, 0.5 ± 0.1 (n = 4); K265A, 8.8 ± 0.8 (n = 6); and K265W, 3.0 ± 0.4, (n = 6) (Mean ± SE). One-way ANOVA, *p* < 0.0001. *B*, Western blot analysis of lysates of HEK293T cells cotransfected with Flag-tagged PDE6C and Pγ (lane 1) or with Flag-tagged PDE6C, Pγ, and HA-tagged AIPL1 (lanes 2–4) using anti-Flag and anti-HA antibodies. β-actin served as a loading control. AIPL1, aryl hydrocarbon receptor-interacting protein-like 1; DMAG, 17-dimethylaminoethylamino-17-demethoxygeldanamycin; PDE6, Phosphodiesterase 6.

To investigate the role of HSP90 in PDE6 maturation, we first tested the effects of HSP90 inhibitor DMAG in a heterologous expression system based on cotransfection of HEK293T cells with PDE6C, Pγ, and AIPL1 (39). In this system, the very low rate of cGMP hydrolysis in the lysates of HEK293T cells transfected with the Flag-PDE6C and EGFP-Pγ is not different from that in untransfected controls, but the cotransfection with the HA-tagged AIPL1 leads to robust PDE6 activity, provided that the inhibitory Pγ subunit is removed with trypsin treatment (Fig. 2*A*) (18). The protein levels of PDE6C are comparable in the presence or absence of AIPL1 (Fig. 2*B*), indicating that AIPL1 induces folding of at least a fraction of the enzyme to its active form.

We used low concentrations of DMAG, 35 and 100 nM, that had no effect on the growth and viability of transfected HEK293T cells. 17-dimethylaminoethylamino-17-demethoxygeldanamycin potently inhibited the expression of PDE6C activity in a dose-dependent manner, whereas the protein levels of the enzyme were not significantly altered (Fig. 2, *A* and *B*). The DMAG treatment also decreased proteolytic stability of AIPL1 in transfected HEK293T cells, as evidenced by the appearance of the ∼30 kDa proteolytic fragment (Fig. 2*B*). Thus, AIPL1 is proteolytically most stable when HSP90 is uninhibited, thereby supporting the interaction of AIPL1 with endogenous HSP90 in transfected HEK293T cells.

### Disruption of the AIPL1 interaction with HSP90 impedes maturation of PDE6

We next examined the role of AIPL1 interaction with HSP90 in inducing PDE6C activity in the heterologous expression system. Because the truncation of the TPR helix 7 extension diminished the binding of AIPL1 to HSP90_AMPPNP_ (Fig. S1), we tested the chaperone activity of AIPL1_1–316_ first. This mutant completely failed to induce cGMP hydrolysis in HEK293 cells cotransfected with PDE6C and Pγ (Fig. 2*A*). Counterparts of K265 in different TPR-domain containing proteins make critical contacts with the C-terminal signature motif MEEVD of HSP90 (40). We introduced two substitutions in AIPL1, K265A, and K265W, that are predicted to disrupt these contacts (Fig. S1*A*). The chaperone activities of AIPL1-K265A and K265W toward PDE6C in the presence of Pγ in HEK293T cells were markedly reduced (Fig. 2*A*) (Fig. 2*A*). Both mutations, K265A and K265W, also disrupted the binding of AIPL1 to HSP90_AMPPNP_ (Fig. S1*B*). Consistent with the stabilizing effect of HSP90 binding on AIPL1, a minor ∼30 kDa proteolytic fragment was observed for all of the three tested mutants, AIPL1_1–316_, K265A, and K265W (Fig. S4). Thus, the chaperone complex for PDE6 minimally comprises HSP90 and AIPL1, and the TPR helix 7 extension as well the core TPR contacts with the C-terminal MEEVD motif of HSP90 are essential for the AIPL1/HSP90 coupling and PDE6 maturation.

### Normal maturation and function of rod PDE6 lacking farnesylation in mouse photoreceptors

**Figure 3:**
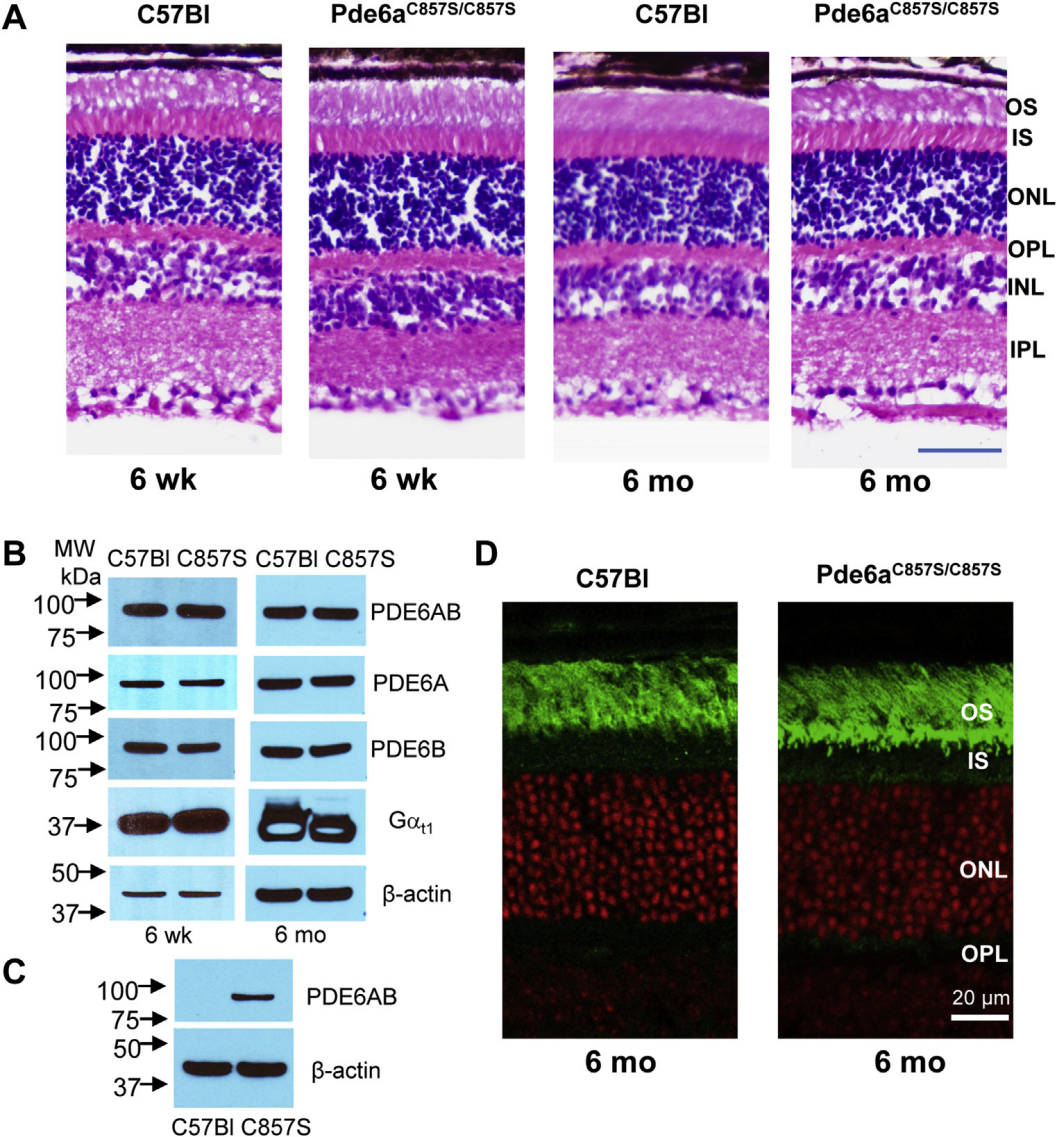
**Expression and trafficking of rod PDE in the retina of a mouse lacking farnesylation of the PDE6A subunit.***A*, retina morphology of 6-weeks and 6-month old Pde6a^C857S/C857S^ and control C57Bl mice. Retina cryosections were stained with H&E. The bar represents 50 μm. *B*, Western blot analysis of PDE6 and transducin-α (Gαt1) expression in retinas of 6-weeks and 6-month-old Pde6a^C857S/C857S^ and control C57Bl mice. Antibodies recognizing both PDE6A and PDE6B antibodies or subunit specific PDE6A and PDE6B antibodies were used. β-actin served as a loading control. *C*, isotonic extracts of retinas from 6-week-old Pde6a^C857S/C857S^ and control C57Bl mice were analyzed by Western blotting with PDE6AB antibodies. The presence of PDE6 in the isotonic extract of Pde6a^C857S/C857S^ retina indicates reduced membrane affinity of farnesyl-less PDE6. *D*, retina cryosections were stained with PDE6B antibodies (*green*). TO-PRO3 nuclear stain – *red*. Immunofluorescence localization of PDE6 in 6-month-old Pde6a^C857S/C857S^ and control C57Bl mice demonstrates proper trafficking the farnesyl-less rod PDE6 to the OS compartment. INL, inner nuclear layer; IPL, inner plexiform layer; IS, inner segments; ONL, outer nuclear layer; OPL, outer plexiform layer; OS, outer segments; PDE6, Phosphodiesterase 6.

Given the apparent 2:1 stoichiometry of the HSP90_AMPPNP_/AIPL1 interaction, we hypothesized that a single prenyl moiety on PDE6 may suffice for anchoring of the client to the chaperone complex and client maturation. Alternatively, maturation of nascent PDE6 may require two HSP90 dimer/AIPL1 complexes, each anchored to a catalytic subunit of the client. To probe these models *in vivo*, we generated a CRISPR/cas9 knock-in mouse with the PDE6A-C857S mutation that abrogates the site for PDE6A farnesylation (Fig. S5). We examined retinal morphology, protein levels of PDE6 and activity, and subcellular localization of the enzyme in mutant Pde6a^C857S/C857S^ and control age-matched C57Bl mice. The retinal morphology of the Pde6a^C857S/C857S^ mice appeared normal by Optical Coherence Tomography imaging of live animals (Fig. S6) and from H&E-staining of retina sections (Fig. 3*A*). The levels of the PDE6A and PDE6B proteins were similar to those in control mice (Fig. 3*B*). The dual prenylation of PDE6 provides for its photoreceptor disc membrane attachment under isotonic conditions (4, 41). Isotonic extract from Pde6a^C857S/C857S^ retinas contained much more PDE6 than that from C57Bl mice, suggesting that PDE6 with only the geranylgeranyl modification has reduced affinity for the membrane (Figs. 3*C* and S7). Immunofluorescence analysis of retina sections from Pde6a^C857S/C857S^ retinas revealed that, despite its reduced membrane affinity, the PDE6 mutant was predominantly targeted to the outer segment compartment with only a minor fraction present at the inner segment/outer segment border, that is, at or near the connecting cilium (Fig. 3*D*).

**Figure 4:**
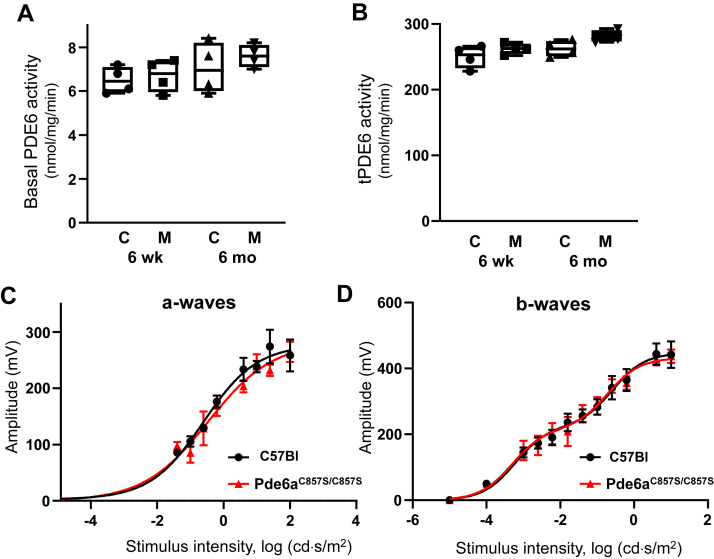
**Normal PDE6 activity and ERG-responses in Pde6a**^**C857S/C857S**^**mice.***A* and *B*, basal and total PDE activities (as represented by trypsin activated PDE activity (tPDE)) are comparable between Pde6a^C857S/C857S^ (M) and control C57Bl (C) mice at ages of 6 weeks and 6 months. *Whiskers* represent minimum and maximum. *Boxes* represent interquartile range. *Line* represents the median and *dots* represent data points. The PDE6 activity values are (nmol•min^−1^•mg^−1^): *A*, 6 weeks: C, 6.5 ± 0.3; M, 6.7 ± 0.4; 6 months: C, 7.0 ± 0.6; M, 7.6 ± 0.3 (Mean ± SE, n = 4); *B*, 6 weeks: C, 251 ± 8; M, 262 ± 4; 6 months: C, 261 ± 6; M, 280 ± 5 (Mean ± SE, n = 4). There were no statistically significant differences between age-matched mutant and control mice (*t* test *p* value > 0.05). *C* and *D*, scotopic ERG a-wave amplitudes and b-wave amplitudes measured from recordings of dark-adapted 6-week-old mice. Points represent the Mean ± SEM (n = 6, *left* and *right eyes* from three mice of each genotype). *Curves* represent fits from single (*C*) or double (*D*) sigmoidal functions. For each flash strength, there were no statistically significant differences (*p* value > 0.05) between Pde6a^C857S/C857S^ and control C57Bl mice. PDE6, Phosphodiesterase 6.

The functional properties of the mutant PDE6 and its ability to support phototransduction were assessed in PDE6 activity assays and by recording scotopic ERG responses. Basal PDE6 activity was assessed in retina homogenates obtained from dark-adapted mice, and the maximal PDE6 activity was measured after treatment with trypsin to selectively remove the inhibitory Pγ subunit (42). Both the basal and the maximal PDE6 activities in Pde6a^C857S/C857S^ and control C57Bl retina homogenates were comparable (Fig. 4*A*). Likewise, the a- and b-wave ERG responses of the mutant and control mice were comparable (Fig. 4*B*), indicating overall normal phototransduction in photoreceptor cells and proper signaling to downstream rod bipolar cells.

### Expression of active PDE6 lacking prenylation

**Figure 5:**
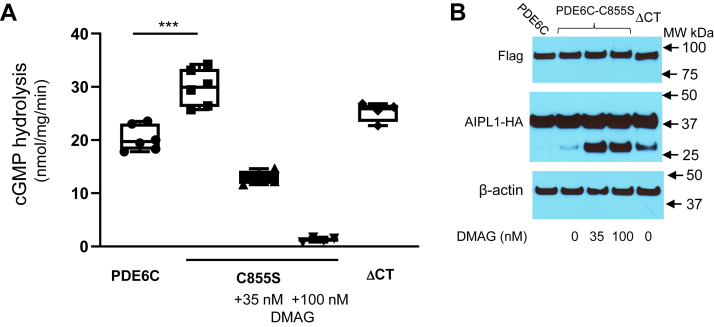
**Maturation of mutant prenyl-less PDE6 in a heterologous expression system.***A*, cGMP hydrolysis in lysates of HEK293T cells cotransfected with AIPL1, Pγ, PDE6C or its prenyl-less mutants C855S and ΔCT (where the indicated HSP90 inhibitor DMAG was present). The mean PDE activity values are (nmol•min^−1^•mg^−1^): PDE6C, 20.3 ± 1.0 (from Fig. 2*A*); PDE6C-C855S, 30.0 ± 1.4 (n = 6); C855S + 35 nM DMAG, 12.9 ± 0.6 (n = 4); +100 nM DMAG, 1.3 ± 0.2 (n = 4); and PDE6CΔCT, 25.3 ± 1.0 (n = 4). One-way ANOVA, *p* < 0.0001. Unpaired *t* test C855S *versus* PDE6C, *p*∗∗∗=0.0002. *B*, Western blot analysis of lysates of HEK293T cells cotransfected with Flag-tagged PDE6C or mutant, Pγ, and HA-tagged AIPL1 using anti-Flag and anti-HA antibodies. β-actin served as a loading control. AIPL1, aryl hydrocarbon receptor-interacting protein-like 1; DMAG, 17-dimethylaminoethylamino-17-demethoxygeldanamycin; PDE6, Phosphodiesterase 6.

Normal maturation of PDE6 lacking farnesyl modification in mouse rods suggested that either a single prenyl moiety is sufficient for the HSP90/AIPL1 complex to bind to and induce active conformation of the enzyme or that the chaperone complex can produce such a conformational change in the absence of the client prenylation. To explore the later possibility, we coexpressed the PDE6C mutant C855S lacking the prenylation site with AIPL1 and Pγ in HEK293T cells. Remarkably, the expressed activity level of PDE6C-C855S was even moderately higher than that for PDE6C controls (Fig. 5*A*). Similarly to the wt PDE6C, maturation of PDE6C-C855S was sensitive to the HSP90 inhibitor DMAG (Fig. 5*A*).

About 30 C-terminal residues linking the core catalytic domain of PDE6 and the prenylation site are not strongly conserved, and the C-termini are flexible and/or unstructured as indicated by the cryo-EM structure of rod PDE6 (43). Coexpression of the PDE6C mutant with truncation of 28 C-terminal residues including the CAAX-box (PDE6CΔCt) with Pγ and AIPL1 in HEK293T cell demonstrated that the flexible C-termini are not required for the chaperone activity of AIPL1/HSP90. The level of cGMP hydrolytic activity of PDE6CΔCt was comparable to that of WT PDE6C (Fig. 5*A*).

The lack of prenylation or the C-terminal truncation of PDE6 also resulted in the appearance of the 30 kDa AIPL1 fragment in the lysates of cotransfected cells, and the abundance of this fragment was markedly elevated in the presence of DMAG (Fig. 5*B*). Thus, despite the reduced stability of the ternary complex with unprenylated PDE6, the HSP90/AIPL1 complex was an effective chaperone for such a client.

### Maturation of PDE6 in the presence of an AIPL1 mutant deficient in prenyl binding

**Figure 6:**
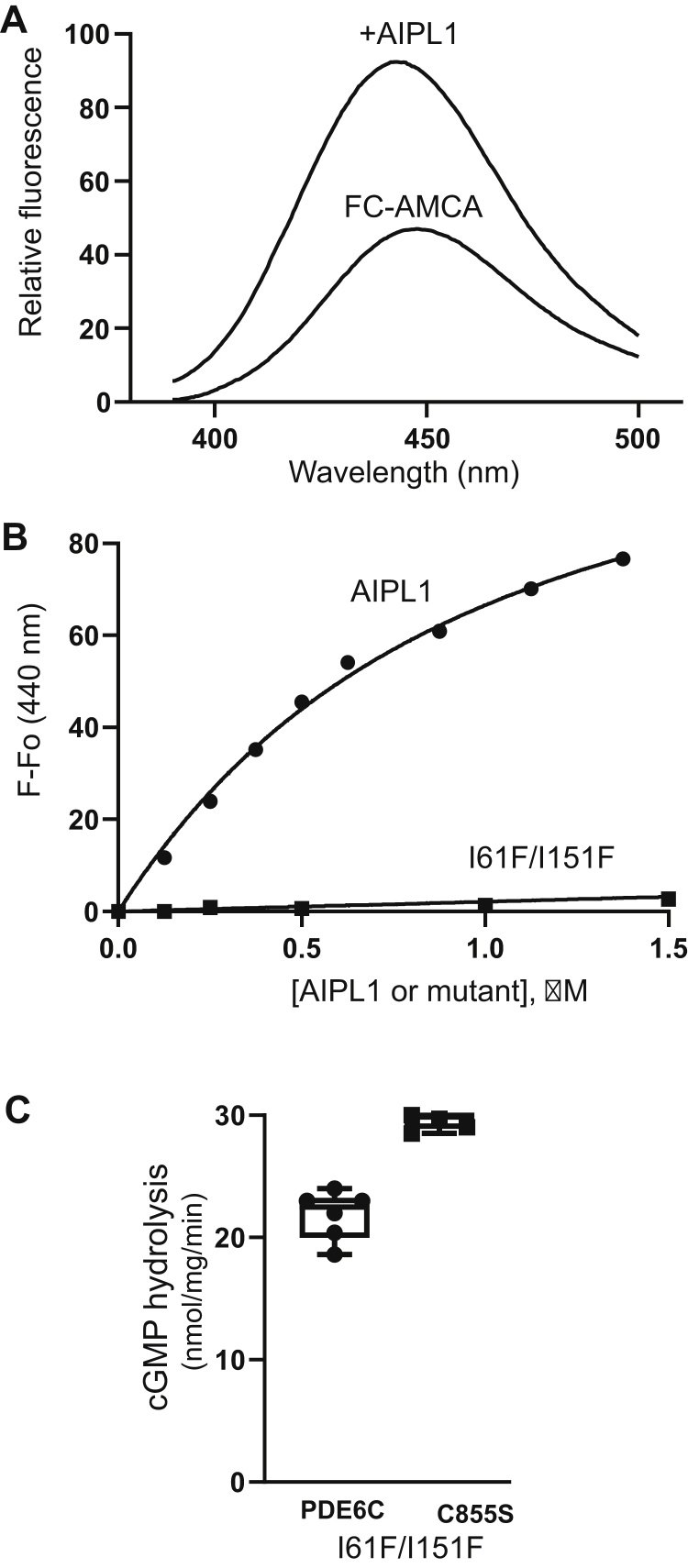
**Prenyl binding by AIPL1 is not absolutely required for PDE6 maturation.***A*, emission spectrum of farnesyl-Cys-AMCA (FC-AMCA) alone (500 nM) and in the presence of 0.5 μM AIPL1 (λ_ex_ =350 nm). *B*, increases in FC-AMCA fluorescence in the presence of AIPL1 or the I61F/I151F mutant were plotted as a function of protein concentration and fit with an equation for binding with ligand depletion. The results of a representative experiment are shown. From three similar experiments, the K_D_ value for AIPL1 is 0.7 ± 0.1 μM (Mean ± SE); for I61F/I151F - ND. *C*, cGMP hydrolysis in lysates of HEK293T cells cotransfected with Pγ, the I61F/I151F mutant of AIPL1, and PDE6C or the C855S mutant of PDE6C. *Whiskers* represent minimum and maximum. *Boxes* represent interquartile range. *Line* represents the median and *dots* represent data points. AIPL1, aryl hydrocarbon receptor-interacting protein-like 1; PDE6, Phosphodiesterase 6.

The ability of the HSP90/AIPL1 complex to chaperone PDE6 with a single prenyl lipid or lacking the prenylation modifications entirely raised a question of whether prenyl binding by AIPL1 is required for proper maturation of the dually prenylated enzyme. To address this question, we generated AIPL1 mutations designed to impair prenyl binding. Residue I61 from the core AIPL1-FKBP is located at the opening of the hydrophobic prenyl-binding pocket, whereas L100 and L104 are within the FKBP “insert” region that acts as a lid over the prenyl-binding site (25). Individual substitutions of one of these residues by a charged residue (I61D, L100D, and L104D) or a polar residue (I61Q) did not alter the protein expression levels (Fig. S8*A*). Mutant AIPL1 proteins, I61D, L100D, and L104D, completely failed to chaperone both PDE6C and PDE6C-C855S (Fig. S8*B*), suggesting destabilization of the hydrophobic FKBP core and/or the dynamics of the “insert” region. The chaperone activity of I61Q was markedly impaired compared to that of AIPL1 (Fig. S8*B*). Steric occlusion of the prenyl-binding site was pursued next by substituting I61 and I151 with a bulky Phe residue. The ability of the I61F/I151F mutant to interact with prenyl moiety was examined in the fluorescence binding assay using farnesyl-Cys-AMCA as a probe. This assay revealed a severe impairment of prenyl binding by the mutant AIPL1 (Fig. 6, *A* and *B*). Unexpectedly, I61F/I151F was a fully competent cochaperone of both PDE6C and PDE6C-C855S in the heterologous expression system (Fig. 6*C*).

## Discussion

The clinically important mechanism of AIPL1-dependent maturation of nascent PDE6 from a nonfunctional conformation into the active native state is yet to be elucidated. Our study advances the understanding of PDE6 maturation in several respects. We provide direct evidence that this process depends on HSP90 and requires the complex between HSP90 and AIPL1. We show that an HSP90 inhibitor DMAG and AIPL1 mutations disrupting its interaction with HSP90 block the maturation of heterologously expressed PDE6. These data are consistent with a large body of evidence for the roles of complexes of HSP90 with cochaperones containing FKBP and TPR domains in inducing functional conformations of client proteins. HSP90 proteins can form symmetric and asymmetric complexes with various cochaperones, but the stoichiometry and molecular details of the underlying interactions are often unclear (36, 44). Particularly, FKBP51 has been shown to interact symmetrically with apo HSP90 in the open state at 1:1 stoichiometry (30). However, another recent study suggests that FKBP51 binds preferentially and asymmetrically to the closed state of HSP90 at 1:2 stoichiometry (37). Our results generally agree with the latter study and indicate that AIPL1 binds preferentially to the closed AMPPNP-bound HSP90 state at 1:2 stoichiometry. The binding of AIPL1 to apo HSP90 is weak, and it is likely mediated only by a tethering of the HSP90 C-terminus to the AIPL1-TPR domain. Increased affinity of AIPL1 for HSP90_AMPPNP_ apparently reflects an additional interaction involving the AIPL1-TPR helix 7 extension and the CTD of HSP90_AMPPNP_. Human AIPL1, compared to the mouse isoform, contains a proline-rich region located C-terminally to the TPR helix 7 extension. This region is predicted to be unstructured (Fig. S9). Thus, the helix 7 extensions in human and mouse AIPL1 proteins are similar and significantly shorter than that in FKBP51. Furthermore, several of the HSP90 contact residues of FKBP51 are not conserved in AIPL1 (Fig. S9). Hence, AIPL1 would make fewer contacts with the HSP90 CTD, and the complex of HSP90 with AIPL1 is significantly weaker than that with FKBP51. We have modeled the HSP90/AIPL1 complex based on the cryo-EM structure of the HSP90/FKBP51 complex, where the position of the AIPL1 FKBP domain is guided by the FK2 domain of FKBP51 (37) (Fig. S10). Although such a position of AIPL1-FKBP is highly speculative, it places the α3-helix of insert region in proximity to HSP90, which agrees with a moderate contribution of the insert region to the AIPL1-HSP90 interface. The binding of one AIPL1 molecule to HSP90_AMPPNP_ dimer precludes symmetrical binding of another AIPL1 molecule, which however, can still be tethered to the second HSP90 C-terminal MEEVD motif. This may provide an explanation for the presence of the minor species with a 2:2 stoichiometry.

As a cochaperone of HSP90, AIPL1 is unique in that its FKBP-domain lacks the prolyl peptidyl isomerase activity and instead encloses an unusual prenyl-binding site (25, 31). In contrast to AIPL1-FKBP, the most homologous FKBP domain of AIP is not capable of the prenyl lipid binding (24). The lipid-binding pocket in AIPL1 may have evolved as a mechanism for attachment of the chaperone complex to nascent PDE6. Furthermore, the prenylated C-termini could have been potentially used as levers to translate ATP-dependent conformational changes in the HSP90/AIPL1 complex into a conformational change in PDE6 yielding a mature enzyme. Our analyses of the PDE6 maturation in Pde6a^C857S/C857S^ mice and expression of functional prenyl-less PDE6C in HEK293T cells argue against such a model. Collectively, these analyses demonstrate that the HSP90/AIPL1 complex can interact with and induce functional conformation in PDE6 lacking prenyl modifications and the unstructured C-termini. Hypothetically, prenylation of PDE6 could have obstructed PDE6 maturation, requiring sequestration of the lipids by the AIPL1 FKBP domain. An obstruction of PDE6 maturation by the prenyl modifications could have resulted from premature PDE6 association with the membrane away from cytosolic HSP90 proteins or from unproductive hydrophobic interactions. This also appears not to be the case, as we demonstrated that a mutant AIPL1 deficient in prenyl binding is a capable chaperone for the prenylated PDE6. Nonetheless, prenyl moieties of PDE6 do contribute to the formation of the ternary chaperone/client complex with nascent PDE6, as evidenced by the increased proteolytic stability of AIPL1 when it is coexpressed in HEK293T cells with PDE6C, but not with PDE6C-C855S. Because AIPL1 does not appear to contribute to trafficking of PDE6 (15), a remaining parsimonious explanation for the evolutionary pressure on AIPL1 to gain and retain prenyl-binding capacity is that it may accelerate the rate of PDE6 maturation. Such an acceleration would result from increased affinity of the HSP90/AIPL1 complex for the client, and it may not be detected in the heterologous expression system.

Many important questions remain as to how the HSP90/AIPL1 complex induces transition of PDE6 to the mature functional state, and how the Pγ subunit assists in this process (18, 45). Clearly, there is an interface between the HSP90/AIPL1 complex and nascent PDE6, which is distinct from the prenyl-binding site and is essential to the conformational transformation of PDE6. The recently described interaction of AIPL1 with Pγ adds to the complexity of the process (28, 38). Our model of the HSP90/AIPL1 complex establishes a framework for future studies of the molecular mechanism of PDE6 maturation.

## Experimental procedures

### Plasmids/cloning

The pET15b vector for the expression of full-length AIPL1 and pcDNA3.1 vector for expression of the full-length HA-tagged m AIPL1 and FLAG tag full-length hPDE6 with enhanced GFP-tagged Pγ were described previously (18, 28). The AIPL1-Δα3 (loop deletion) construct was generated by replacing the residues 111 to 132 of AIPL1 with five glycine residues described previously (38).

The DNA sequence encoding the AIPL1-316 (aa 1–316) was PCR-amplified from pCDNA3.1 vector harboring AIPL1 gene (18) and cloned into either the NcoI/NdeI site of the pET15b vector (Novagen), for expression as a His_6_-tagged protein in *E. coli*, or the HindIII/Xba1 sites of pcDNA3.1(+), for expression as an HA-tagged protein in cultured HEK293T cells. Mutations K265A, K265W, I61D, L100D, L104D, I61Q, & double I61F/I151F were introduced into AIPL1 using the standard QuikChange site-directed mutagenesis protocol.

To obtain PDE6CΔCt, 28 C-terminal residues of PDE6C were deleted using the pCDNA3.1 vector harboring PDE6C-IRES-EGFP-Pγ gene (18) and the NEB mutagenesis protocol (https://nebasechanger.neb.com/). PDE6C-C855S was introduced using the standard QuikChange site-directed mutagenesis protocol. A DNA sequence encoding the full-length human HSP90β (residues 1–724) was PCR-amplified from cDNA isolated from HEK293T cells and cloned into the pET15b vector using Nco1-Nde1 sites. To obtain the Avi-tagged HSP90β, HSP90β was cloned into a modified pET21a vector with the N-terminal His6-tag followed by the Avi tag and TEV cleavage site (46).

### Protein purification

The full-length mouse AIPL1 and mutant AIPL1 proteins AIPL1-Δα3 (Δ111–132), AIPL1_1–316_, I61F/I151F, K265A, and K265W were expressed in BL21-(DE3) *E. coli* cells and purified over Ni-NTA resin (EMD Millipore), followed by ion-exchange chromatography on a Mono Q5 column (Bio-Rad), as previously described (28, 47, 48). Final purification step included size-exclusion chromatography (SEC) on a HiLoad 16/600 Superdex 75 column (GE Healthcare) equilibrated against 50 mM Tris·HCl (pH 7.5) buffer containing 5 % glycerol, 200 mM NaCl, and 1 mM tris(2-carboxyethyl)phosphine (TCEP).

Cells for expression of the His_6_-Avi-tagged HSP90β were grown in TB media supplemented with biotin (10 mg/liter), induced at OD_600_ of 0.6 with 250 μM IPTG, and further grown overnight at 16 °C. *In vivo* biotinylation was performed as described (46). The cells were harvested and sonicated on ice (five 30-s pulses) in 50 mM Tris–HCl buffer (pH7.5) containing 100 mM NaCl, 5 % glycerol, and 2.5 mM β-mercaptoethanol (BME) (buffer A), and protease inhibitor mixture (Roche Applied Science). The His_6_-Avi-tagged proteins were purified over Ni-NTA resin (EMD Millipore) using buffer A containing 250 mM imidazole for elution and further purified by Q sepharose (GE healthcare) anion exchange chromatography followed by SEC on a HiLoad 16/600 Superdex 200 column (GE Healthcare) equilibrated against 50 mM Tris·HCl (pH 7.5) buffer containing 5 % glycerol, 200 mM NaCl, and 1 mM TCEP.

### Bio-layer interferometry binding assay

An Octet RED96 system and SA-coated biosensors (FortéBio) were used to measure association and dissociation kinetics for N-terminally biotinylated hHSP90B with AIPL1, AIPL1-Δα3, AIPL1_1–316_, K265A, and K265W. Binding studies were performed in 25 mM Tris, 200 mM NaCl, 2.5% glycerol, 1 mM TCEP, 0.5 mg/ml bovine serum albumin, pH 7.5. All steps were performed at 26 °C, with biosensors stirred into 0.2 ml of sample in each well at 1000 rpm at a data acquisition rate of 5.0 Hz. N-terminally biotinylated HSP90B protein was loaded onto SA sensors at a 50 to 75 μg/ml concentration for 60 to 90 s. The data for association and dissociation phases of the assay were collected as shown in Figure 1, *B* and *C*. To correct for baseline drift and nonspecific binding, reference sensors with bound biotinylated HSP90β were used in the BLI assays without additions of AIPL1 proteins. Kinetic data fitting was performed using FortéBio Data Analysis software 10.0. For each concentration of AIPL1 or mutants, dissociation rate constant (k_off_) values were calculated from the corresponding dissociation phases of the curves. These k_off_ values were used to calculate the association rate constant (k_on_) values from the association phases for each concentration according to the equation: k_on_= k_observed_-k_off_/[AIPL1 or mutant].

The average k_on_ and k_off_ were calculated as means of the individual k_on_ and k_off_ values for all curves. The affinity constant K_D_ was calculated as mean k_off_/mean k_on_ or from fitting steady-state data to the equation for one site-specific binding using the GraphPad Prism 8 software.

### Phosphodiesterase 6 activity assays

For assays of PDE6 activity in HEK293T cell lysates, the cells were cultured and maintained in DMEM containing 10% fetal bovine serum (Gibco), transfected with plasmids encoding human PDE6C (or mutants), mouse Pγ, and mouse AIPL1 (or mutants) (1 μg each) using FuGene6 (Promega) according to manufacturer’s instructions. The lysates from HEK293T cells were prepared 48 h posttransfection by sonication with two 4-s pulses in isotonic buffer (20 mM Tris–HCl (pH 7.5), 120 mM KCl, 1 mM MgCl_2_, and 1X Protease inhibitor cocktail (EDTA-free, Roche). Protein concentrations were measured using Bradford Assay. The samples were treated with 0.1 mg/ml TPCK-Trypsin (Sigma) on ice for 10 min to selectively degrade Pγ, after which trypsin was inhibited with the addition of 10-fold excess of soybean trypsin inhibitor (Sigma). Cell lysates (protein concentration ∼5 mg/ml) were diluted 4 to 600 fold into 40 μl (final volume) of 20 mM Tris–HCl (pH 7.5) buffer containing 120 mM NaCl, 2 mM MgSO4, 1 mM BME, 0.1 U bacterial alkaline phosphatase, and 10 μM [^3^H]cGMP (100,000 cpm) (PerkinElmer) for 15 min at 37 °C. The reaction was stopped by adding AG1-X2 cation exchange resin (0.5 ml of 20% bed volume suspension). The samples were incubated for 6 min at 25 °C with occasional mixing and spun at 10,000g for 3 min. 0.25 ml of the supernatant was removed for counting in a scintillation counter.

For the assays of PDE6 activity in retina homogenates, for each genotype at two ages (6 weeks and 6 months old), two mouse retinas were homogenized by sonication (two 5-s pulses) in 120 μl of 20 mM Tris–HCl buffer (pH 7.5) containing 120 mM NaCl, 1 mM MgSO_4_, and 1 mM BME. After brief centrifugation (20,000*g*, 2 min, 4 ^°^C) to remove cell debris, retinal homogenates (typically, 5–6 mg protein/ml) were used to measure basal PDE6 activities with final dilutions of 1:150 to 1:200 based on protein concentration. Maximal (trypsin-activated) PDE6 activities were measured from retinal homogenates treated with trypsin (100 μ/ml) for 10 min at 25 ^°^C. Trypsin treatment was terminated by the addition of 10X soybean trypsin inhibitor (Sigma) and incubation for 5 min at 25 ^°^C, followed by centrifugation at 20,000*g* for 3 min at 4 ^°^C. The final dilutions of trypsin-treated retinal homogenates in the assays of maximal PDE6 activity were 1:4000 to 1:6000, and the activity assays were carried out as above.

### Cross-linking

HSP90β and AIPL1 proteins were purified by SEC in 25 mM Hepes (pH7.5) with 200 mM NaCl, 5 % Glycerol, and 1 mM TCEP. Cross-linking reactions were initiated with the addition of DSS (0.5 mM final concentration) or glutaraldehyde (0.03%) to HSP90β (15 μM), AIPL1 (25 μM), or a mixture of HSP90β with AIPL1 and were allowed to proceed for 15 to 30 min at 25 °C after which they were quenched with the addition of 30 mM Tris–HCl (pH 7.5) (final concentration). Cross-linked proteins were resolved on 3 to 8 % NuPage-Tris Acetate gel.

### Mass photometry

Mass Photometry experiments were performed on a Refeyn TwoMP (Refeyn Ltd) (49). No.1.5, 24 mm × 50 mm microscope coverslips (Thorlabs Inc) were cleaned by serial rinsing with Milli-Q water and HPLC-grade isopropanol (Sigma Aldrich), on which a CultureWell gasket (Grace Bio-labs) was then placed. All measurements were performed at 25 °C in Dulbecco's phosphate-buffered saline without calcium and magnesium (Thermo Fisher). For each measurement, 15 μl of Dulbecco's phosphate-buffered saline buffer was placed in the well for focusing, after which 3 to 5 μl of 100 nM protein was introduced and mixed. Movies were recorded for 60 s at 50 fps under standard settings. Mass Photometry measurements were calibrated using protein standard mixture: β-Amylase (56, 112 and 224 kDa) and Thyroglobulin (670 kDa). Mass Photometry data were processed using DiscoverMP (Refeyn Ltd).

Generation of Pde6a^C857S/C857S^ mice is described in Supporting information text and Fig. S5. All experimental procedures involving the use of mice were performed in accordance with the National Institutes of Health guidelines and the protocol approved by the University of Iowa Animal Care and Use Committee.

### Antibodies

Anti PDE6AB and PDE6A antibodies were obtained as described (50). The following commercial antibodies were used: anti-PDE6B PA1-722 (ThermoFisher), anti-FLAG and anti-rod Gα_t1_ K-20 (Santa Cruz Biotechnology), anti-HA (BioLegend), and anti β-actin (Cell Signaling Technology). Procedures for immunoblotting, retinal morphology, and immunofluorescence were performed as previously described (50, 51).

### Electroretinography

Electroretinography recordings were obtained for dark-adapted mice using the Espion E3 system (Diagnosys LLC) essentially as described previously (52). Electroretinography responses were evoked in mice by a series of flashes ranging from 0.0001 to 100 cd s/m^2^. Responses to six sweeps were averaged for dim flashes up to 0.6 cd s/m^2^, two sweeps were averaged for 4 cd s/m^2^, and responses to brighter flashes were recorded without averaging. Intersweep intervals for flashes with increasing strength were increased from 10 to 60 s to allow full recovery from preceding flashes.

### Optical coherence tomography

Mice were injected with a standard mixture of ketamine/xylazine (intraperitoneal injection of (100 mg ketamine +10 mg xylazine)/kg body weight (KetasetÒ, Fort Dodge Animal Health; AnaSedÒ, Lloyd Laboratories). Upon anesthesia, eyes were hydrated with balanced salt solution (Alcon Laboratories). To obtain retinal images, the tear film was wicked away and eyes were imaged with a Bioptigen spectral domain optical coherence tomographer (Bioptigen, Inc) using the mouse retinal bore. The volume intensity projection was centered on the optic nerve. Scan parameters were as follows: rectangular volume scans 1.4 mm in diameter, 1000 A-scans/B-scan, 100 B-scans/volume, 1 frame/B-scan, and 1 volume. After imaging, eyes were hydrated with artificial tears and mice were provided supplemental indirect warmth for anesthesia recovery. Retinal thickness was measured from retinal images within the Bioptigen InVivoVueClinic Software four times per eye using vertical angle-locked B-scan calipers from the internal limiting membrane to the retinal pigment epithelium. The six retinal thickness measurements obtained from two eyes of each animal were averaged together to produce a single retinal thickness measurement per animal. All retinal thickness measurement values are reported as an average ± SD.

### Fluorescence binding assay

Labeling of S-farnesyl-L-cysteine FC with AMCA (6-((7-amino-4-methylcoumarin-3-acetyl)amino) hexanoic acid, succinimidyl-ester) was performed as described previously (24). Emission spectra of FC-AMSA (500 nM) in the absence or presence of increasing concentrations of AIPL1 or I61F/I151F were recorded with excitation at 350 nm using F-2700 fluorescence spectrophotometer (Hitachi), and the changes in fluorescence at 440 nm were plotted and fitted with an equation for binding with ligand depletion the GraphPad Prism 8 software.

### Statistical analyses

Unless otherwise indicated, measurements were taken from at least three independent experiments, and the data are shown as mean value and standard error. The data are plotted in a box and whiskers type of graph, where whiskers represent minimum and maximum, boxes represent interquartile range, line represents the median, and dots represent data points. Measurements were compared using *t* test and one-way ANOVA.

## Data availability

All data are contained within the article or the Supplementary Information.

## Supporting information

This article contains supporting information (53).

## Conflict of interest

The authors declare that they have no conflicts of interest with the contents of this article.
