# Supplementary material for: Molecular insights into the maturation of phosphodiesterase 6 by the specialized chaperone complex of HSP90 with AIPL1
Source: J Biol Chem. 2022 Jan 21;298(3):101620. doi: 10.1016/j.jbc.2022.101620 (PMC8857470; doi:10.1016/j.jbc.2022.101620)
Supplement: Supplemental Figures S1–S10 [file mmc1.pdf]

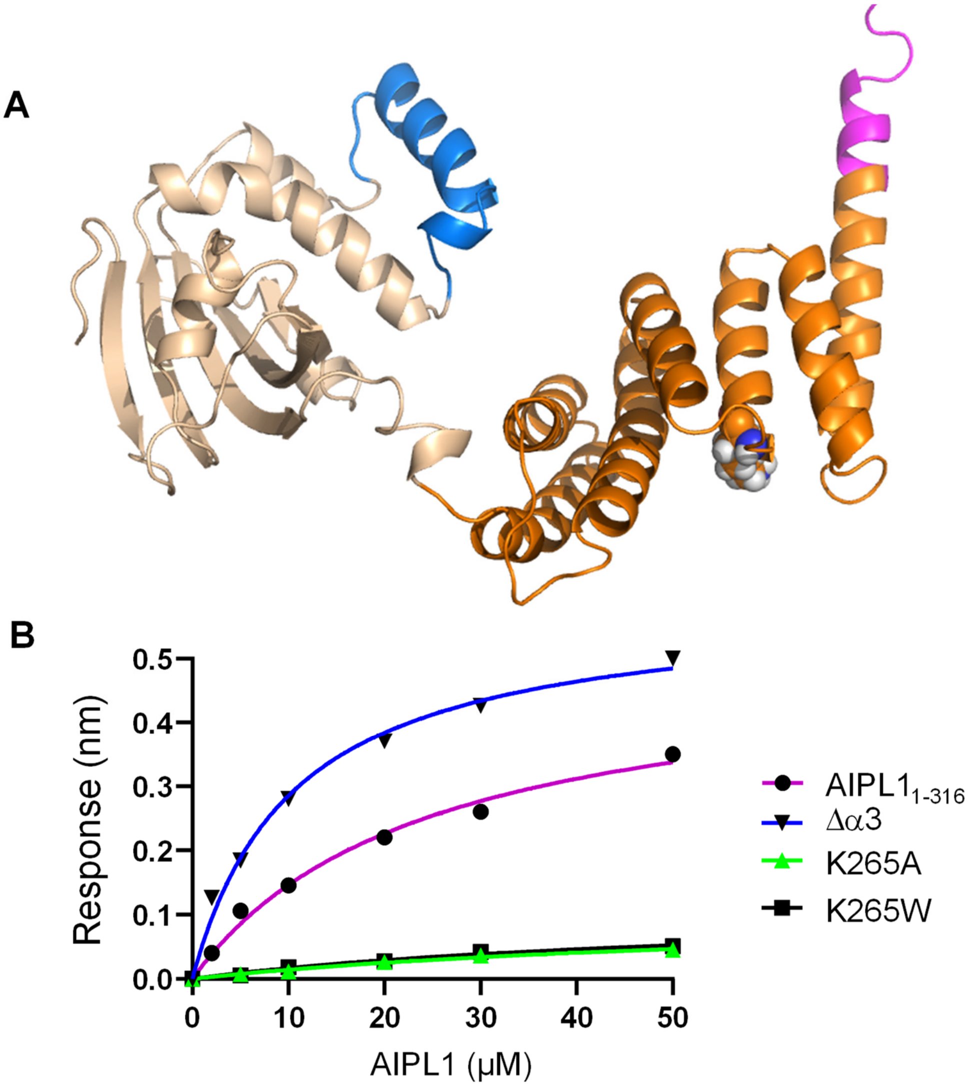

Suppl. Fig. 1. A. A model of mouse AIPL1 highlighting mutations aimed to disrupt the protein binding to HSP90. The positions and orientation of the FKBP domain (PDB ID 5U9I, wheat) and the TPR domain of AIPL1 (PDB ID 6PX0; orange) were guided by the FK2 and TPR domains of FKBP51 from its complex with HSP90 (PDB ID 7L7I). The C-terminal 12 residues of AIPL1 (magenta) were deleted in AIPL1$_{1-316}$; the insert region α3-helix (blue) was replaced by 5 Gly residues in Δα3; K265 is shown as spheres. **B**. The steady state BLI binding curves for mutant AIPL1 proteins and HSP90$_{AMPPNP}$ coupled to a streptavidin biosensor. The results of representative experiments are shown. For n=2 experiments, AIPL1$_{1-316}$ mean K_D=27 µM (range 24-30 µM); Δα3, mean K_D=14 µM (range 11-17 µM); K265A, K265W K_D>50 µM.

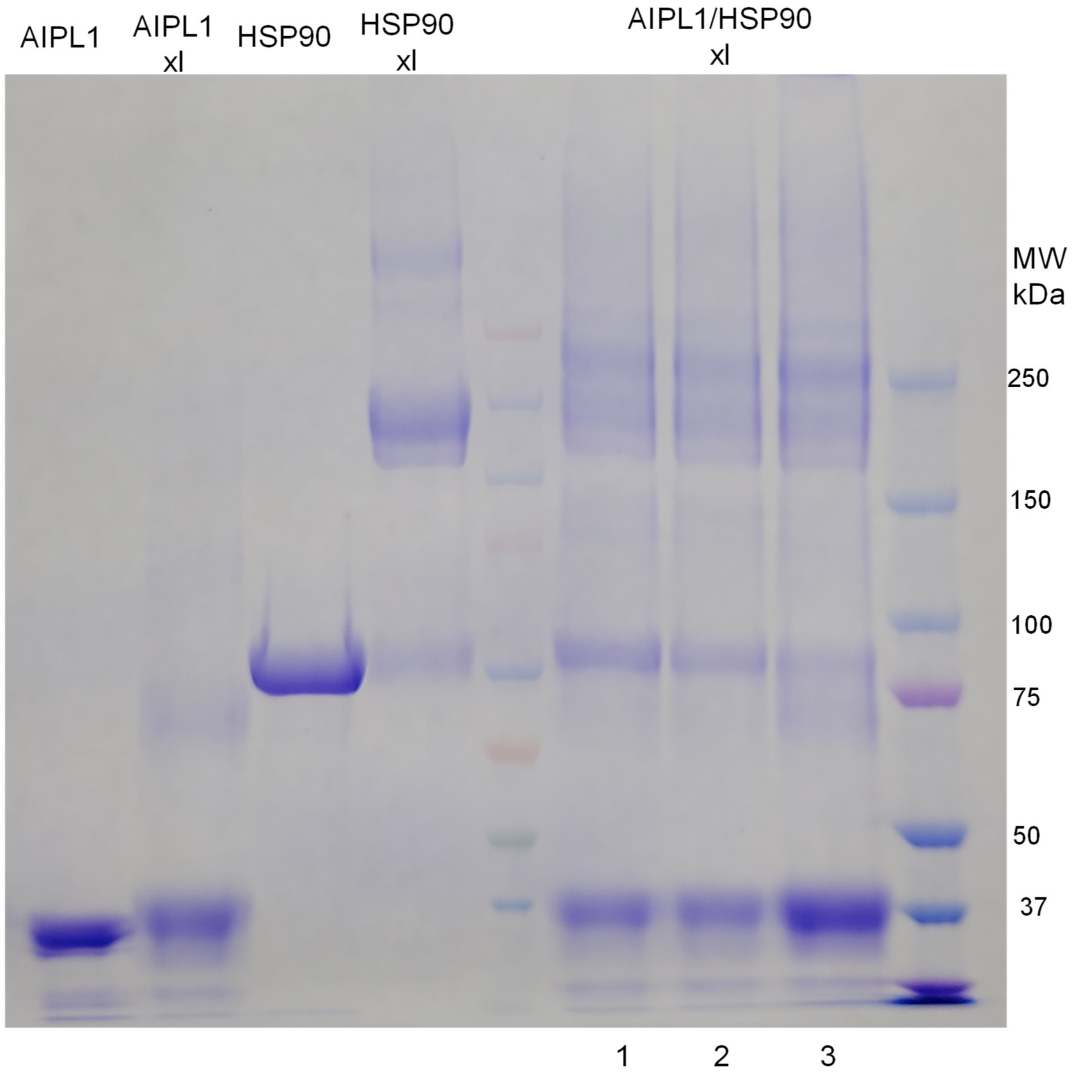

Suppl. Fig. 2. Crosslinking of AIPL1 with HSP90. Coomassie-stained SDS-PAGE gel. AIPL1, HSP90 and AIPL1/HSP90 mixture were crosslinked (xl) with DSS (0.5 mM) for 15 min (**1**) or 30 min (**2**,**3**). (**1**, **2** – 7.5 µM HSP90 dimer, 25 µM AIPL1; **3** – 3.5 µM HSP90 dimer, 15 µM AIPL1).

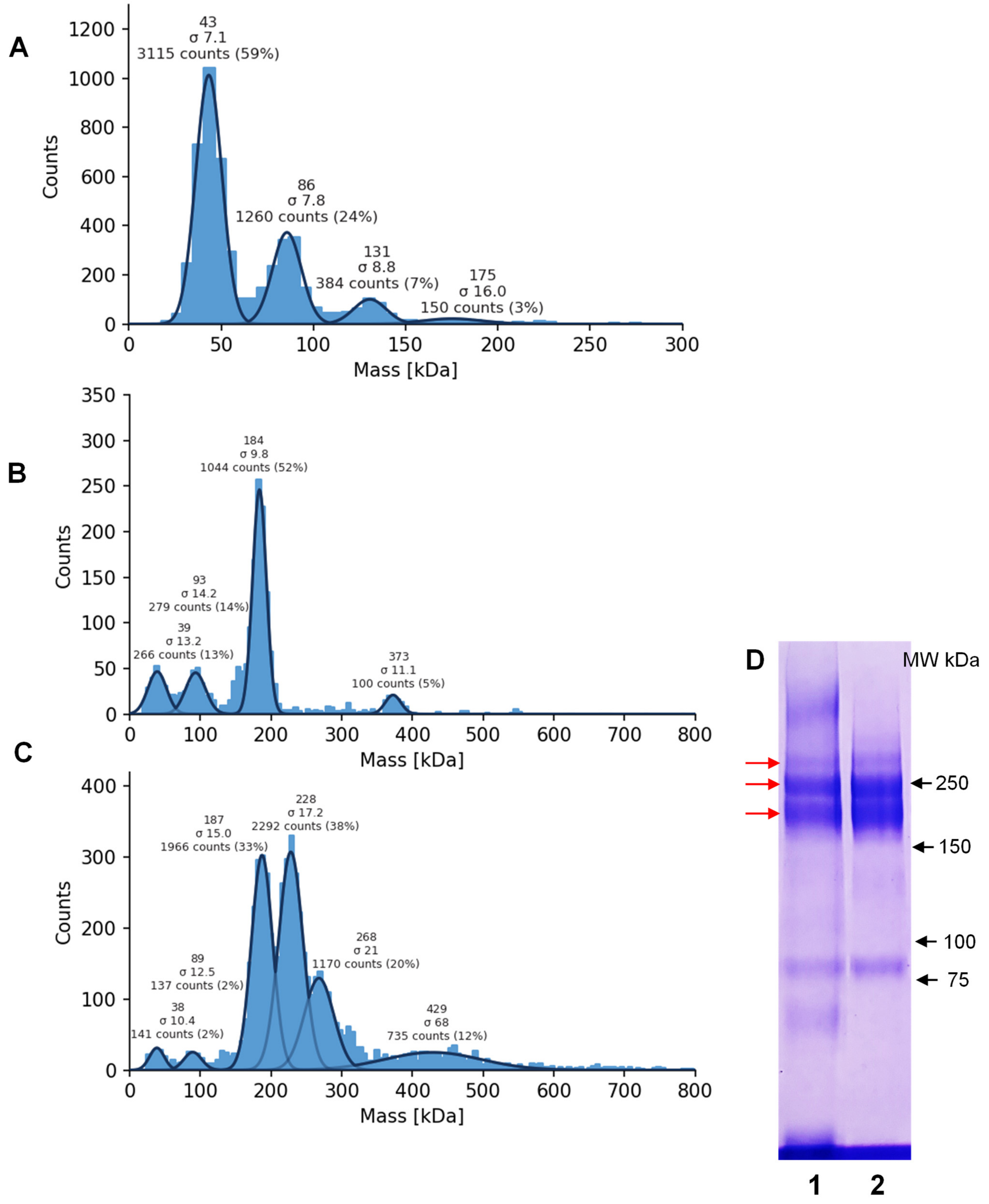

Suppl. Fig. 3. Analysis of crosslinking products of AIPL1 and HSP90 by mass photometry. AIPL1 (25 μM) (**A**), HSP90 (15 μM) (**B**), or the AIPL1/HSP90 mixture (**C**) were crosslinked with glutaraldehyde (0.03 %) for 15 min at 25°C and the crosslinking products were analyzed by mass photometry. Representative histograms are shown. Self-crosslinked HSP90 dimer (B) displays a mass of 184 kDa. The major (228 kDa) and minor (268 kDa) products of AIPL1 crosslinking to HSP90 (C) are not present among the products of self-crosslinking of AIPL1 (A) and HSP90 (B) and correspond to the predicted masses for the complexes containing one molecule and two molecules of AIPL1 per HSP90 dimer, respectively. **D**. Coomassie- stained SDS-gel. Lane 1 - AIPL1/HSP90 mixture crosslinked with glutaraldehyde; lane 2 – peak fraction of the crosslinked AIPL1/HSP90 complex after SEC analyzed by mass photometry in (C). The three major products in (C) migrate at ~210, 250, and 290 kDa (indicated by red arrows).

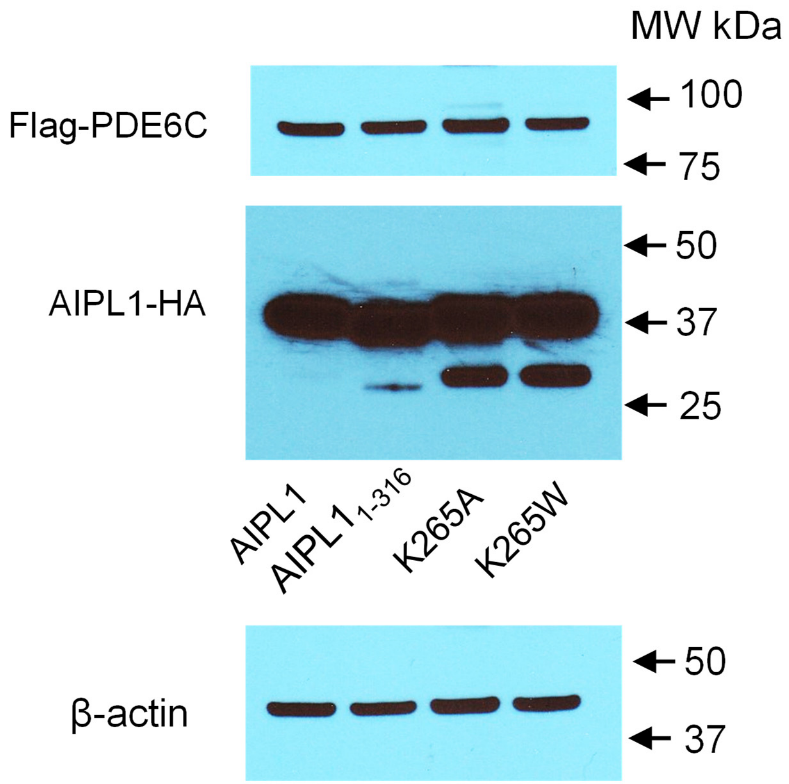

Suppl. Fig. 4. Proteolytic stability of mutant AIPL1 proteins in transfected HEK293T cells. Western blot analysis of lysates of HEK293T cells co-transfected with Flag-tagged PDE6C, Pγ and HA-tagged AIPL1 or AIPL1 mutants using anti-Flag and anti-HA antibodies. β-actin served as a loading control.

Generation of Pde6a$^{C857S/C857S}$ mice Animals

C57BL/6J mice were purchased from Jackson Labs (000664; Bar Harbor, ME). Male mice older than 8 weeks were used to breed with 3-5 week old super-ovulated females to produce zygotes for pronuclear injection. Female ICR (Envigo; Hsc:ICR(CD-1)) mice were used as recipients for embryo transfer. All animals were maintained in a climate-controlled environment at 25C and a 12/12 light/dark cycle. Animal care and procedures conformed to the standards of the Institutional Animal Care and Use Committee of the Office of Animal Resources at the University of Iowa.

Preparation of Cas9 RNPs and the injection mix

Chemically modified CRISPR-Cas9 crRNAs and CRISPR-Cas9 tracrRNA were purchased from IDT (Alt-R® CRISPR-Cas9 crRNA; Alt-R® CRISPR-Cas9 tracrRNA (Cat# 1072532)). The crRNAs and tracrRNA were suspended in T10E0.1 and combined to 1 ug/ul (~29.5 uM) final concentration in a 1:2 (ug:ug) ratio. The RNAs were heated at 98C for 2 min and allowed to cool slowly to 20C in a thermal cycler. The annealed cr:tracrRNAs were aliquoted to single-use tubes and stored at -80C.

Cas9 nuclease was also purchased from IDT (Alt-R® S.p. HiFi Cas9 Nuclease). Cr:tracr:Cas9 ribonucleoprotein complexes were made by combining Cas9 protein and cr:tracrRNA in T10E0.1 (final concentrations: 300 ng/ul (~1.9 uM) Cas9 protein and 200 ng/ul (~5.9 uM) cr:tracrRNA). The Cas9 protein and annealed RNAs were incubated at 37C for 10 minutes. The RNP complexes were combined with single-stranded repair template (ultramer oligo; IDT) and incubated an additional 5 minutes at 37C. The concentrations in the injection mix were 30 ng/ul (~0.2 uM) Cas9 protein and 20 ng/ul (~0.6 uM) cr:tracrRNA and 20 ng/ul single stranded repair template.

Collection of embryos and injection

Pronuclear-stage embryos were collected using methods described in (REF1). Embryos were collected in KSOM media (Millipore; MR101D) and washed 3 times to remove cumulous cells. Cas9 RNPs and single stranded repair template were injected into the pronuclei of the collected zygotes and incubated in KSOM with amino acids at 37C under 5% CO2 until all zygotes were injected. Fifteen to 25 embryos were immediately implanted into the oviducts of pseudo-pregnant ICR females.

Genotyping.

Heterozygous and homozygous Pde6a$^{C857S/C857S}$ mice were genotyped by sequencing 294-bp PCR products obtained using mouse genomic DNA as template and the following primers:

>Pde6a_C857S_PMKI_101 TTCCTCTGCTTCTCCTGACT

>Pde6a_C857S_PMKI_102 CTTCCAAGCTCCTGGTTCTTTA

NM_146086_Mus musculus phosphodiesterase 6A, cGMP-specific, rod, alpha (Pde6a), mRNA

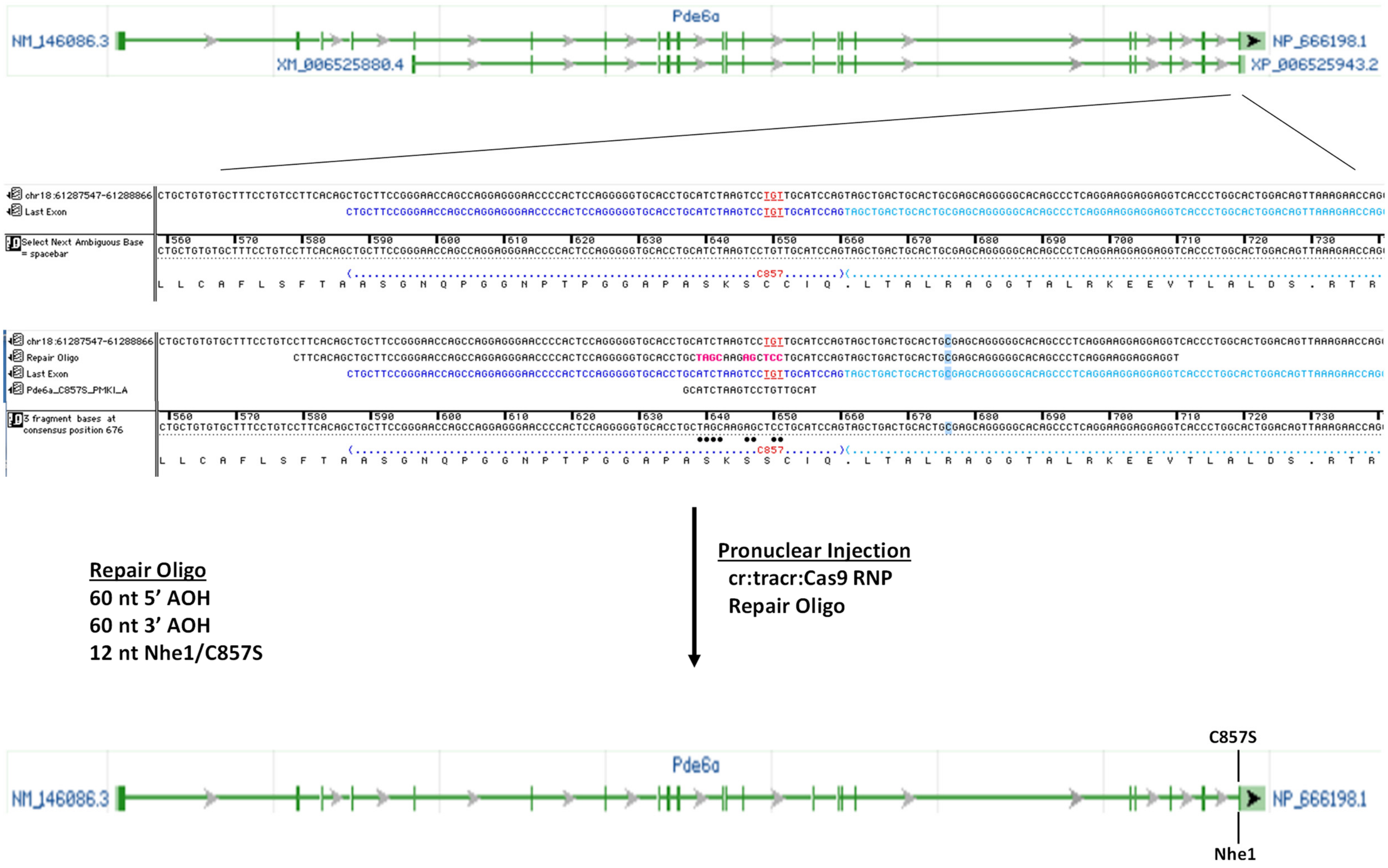

Suppl. Fig. 5. CRISPR/Cas9-based genome editing to introduce the C857S mutation in the mouse *Pde6a* gene.

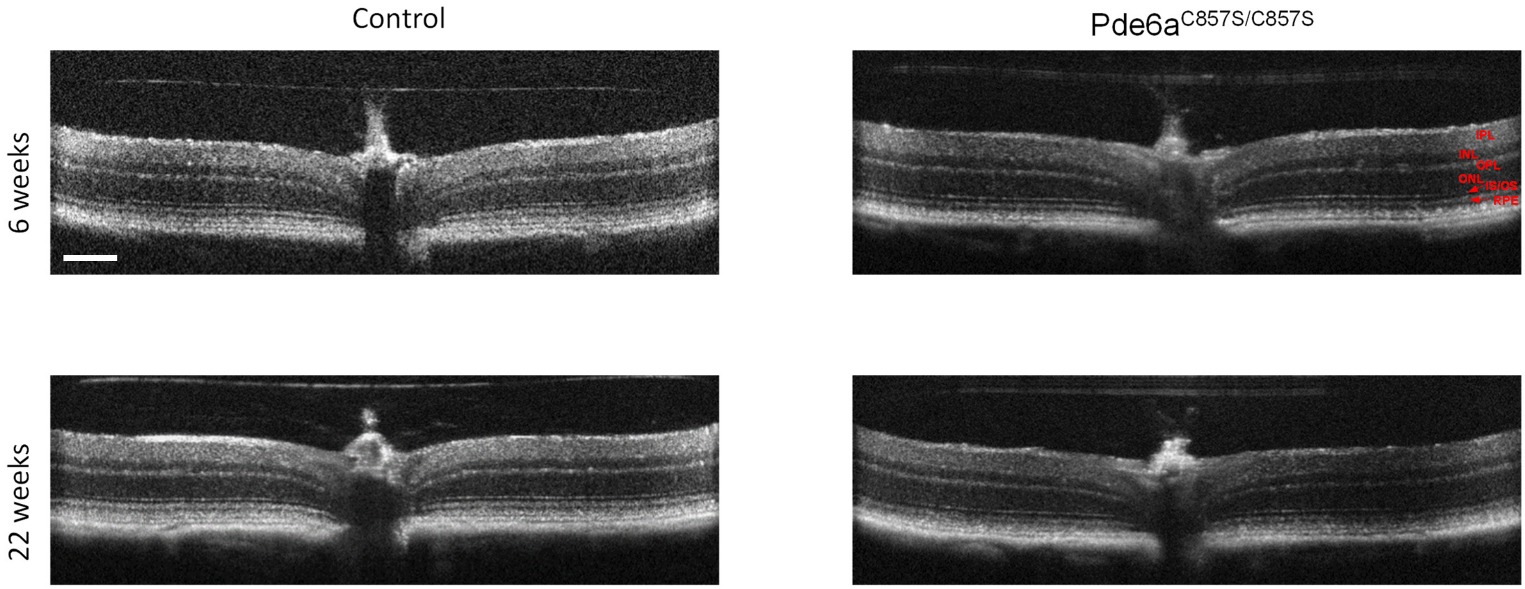

Suppl. Fig. 6. Optical coherence tomography. Retinas appear normal in homozygous Pde6a$^{C857S/C857S}$ mice and their heterozygous littermates at 6 weeks and 22 weeks of age. Measurements of outer retina (outer plexiform layer to retinal pigment epithelium, µm): 6- and 22-weeks controls - 116.6±1.9 and 115.3±3.1; 6 and 22 weeks Pde6a$^{C857S/C857S}$ – 114.0±3.1 and 113.4±2.8. Scale bar – 112.5 µm.

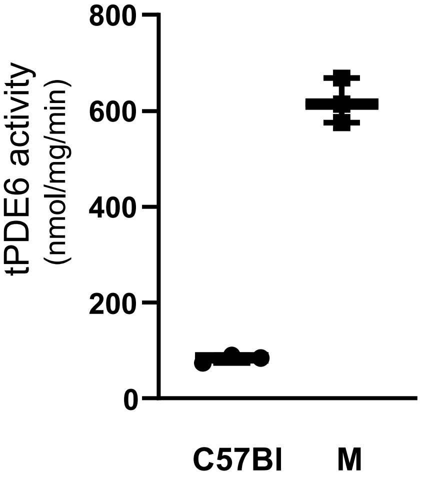

Suppl. Fig. 7. Trypsin activated PDE activities (tPDE) in isotonic extracts of 6-week-old control C57Bl and Pde6a$^{C857S/C857S}$ (M) mice. Whiskers represent minimum and maximum. Boxes represent interquartile range. Line represents the median, and dots represent data points.

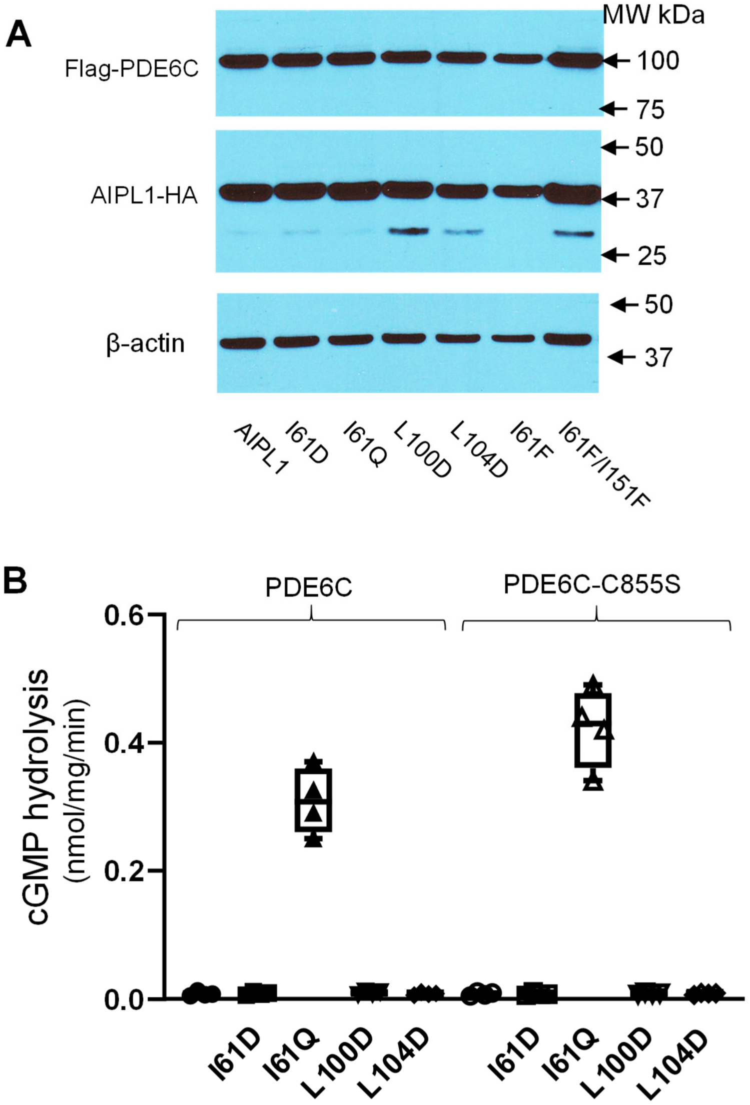

Suppl. Fig. 8. A. Western blot analysis of lysates of HEK293T cells co-transfected with Flag-tagged PDE6C, Pγ, and HA-tagged AIPL1 or its mutants using anti-Flag and anti-HA antibodies. β-actin served as a loading control. **B**. cGMP hydrolysis in lysates of HEK293T cells co-transfected with Pγ, PDE6C or its C855S mutant alone or with AIPL1 mutants. Whiskers represent minimum and maximum. Boxes represent interquartile range. Line represents the median, and dots represent data points (n=4). Trypsin-activated PDE6C activity (nmol•min^{-1}• mg^{-1}) in the presence of I61Q is 0.31±0.03 (n=4) vs 20.3±1.0 in the presence of AIPL1 (from Fig. 2). Trypsin-activated PDE6C-C855S activity in the presence of I61Q is 0.42±0.03 (n=4) vs 30.0±1.4 in the presence of AIPL1 (from Fig. 5).

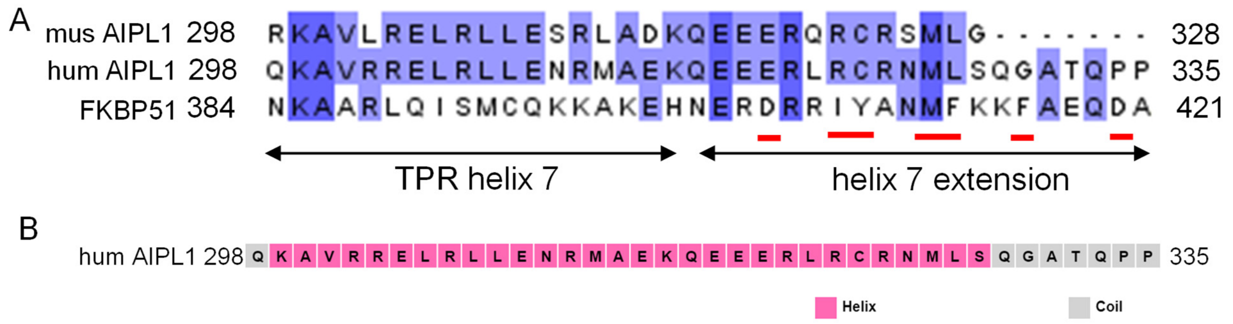

Suppl. Fig. 9. (**A**) Alignment of the TRP helix 7 and helix 7 extensions of mouse and human AIPL1 and human FKBP51. FKBP51 residues making contacts with HSP90 are underlined by red line (based on the PDB 7L7I). Several of the contact residues are missing or not conserved in AIPL1 proteins. (**B**) Residues C-terminal to Ser328 in human AIPL1 are not predicted by PSIPRED to form an α-helix.

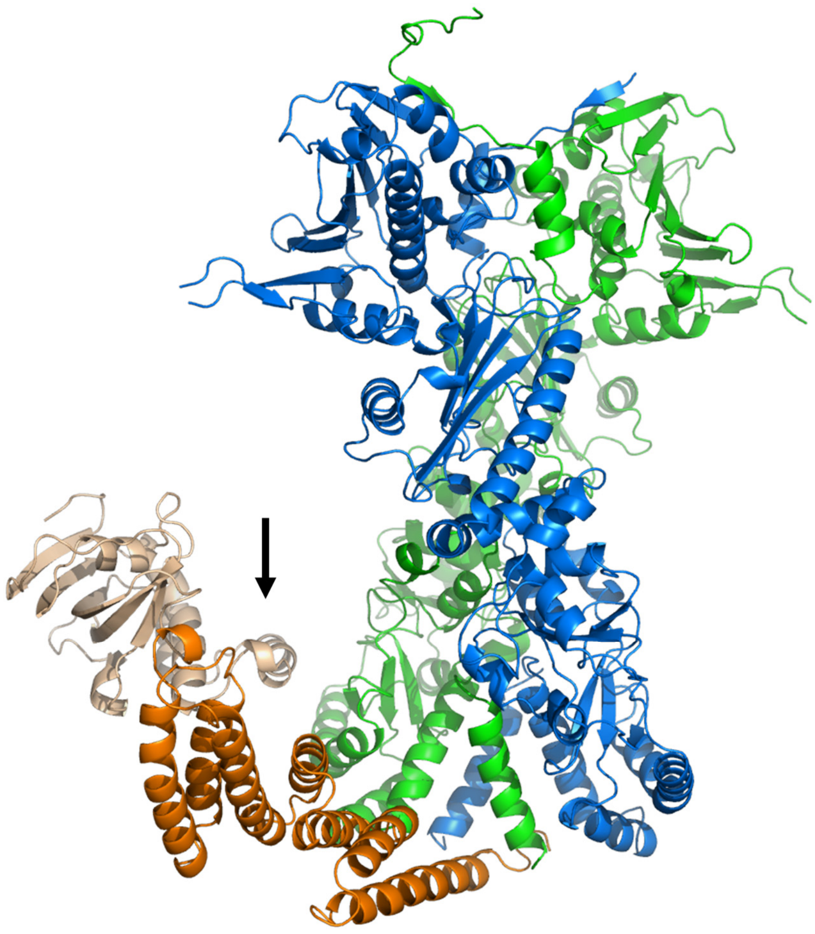

Suppl. Fig. 10. A model of the complex of HSP90 with AIPL1. The positions and orientation of the FKBP domain (PDB ID 5U9I, wheat) and the TPR domain of AIPL1 (PDB ID 6PX0; orange) were guided by the FK2 and TPR domains of FKBP51 from its complex with HSP90 (PDB ID 7L7I, green and blue). In the model, the α3 helix of the AIPL1-FKBP insert region (indicated by arrow) comes into proximity to HSP90.
